# Application of Blended Learning to Veterinary Gross Anatomy Practical Sessions: Students’ Perceptions of Their Learning Experience and Academic Outcomes

**DOI:** 10.3390/ani13101666

**Published:** 2023-05-17

**Authors:** Olga Gómez, Maria García-Manzanares, Deborah Chicharro, Miriam Juárez, Clara Llamazares-Martín, Enrique Soriano, José Terrado

**Affiliations:** Department of Animal Medicine and Surgery, Facultad de Veterinaria, Universidad CEU Cardenal Herrera, CEU Universities, C/Tirant lo Blanch, 7, Alfara del Patriarca, 46115 Valencia, Spain; olgoro@uchceu.es (O.G.); maria.garcia8@uchceu.es (M.G.-M.); debora.chicharro@uchceu.es (D.C.); miriam.juarez@uchceu.es (M.J.); clara.llamazares@ucv.es (C.L.-M.); e.soriano@vetmedia.es (E.S.)

**Keywords:** anatomy, flipped classroom, blended learning, dissection, prosection, team-work

## Abstract

**Simple Summary:**

Blended learning strategies are becoming more popular in health sciences teaching, including veterinary medicine. However, the use of these methods in practical classes is less commonly described. This paper outlines a blended learning approach using flipped classrooms, collaborative learning, and gamification in gross anatomy practicals for first-year veterinary students. The practicals were restructured to include video pre-viewing, quizzes, collaborative group work, and a card game. Results showed a small but significant improvement in practical anatomy exam scores for the locomotor apparatus, with no significant difference in organic systems exams. Student satisfaction was high, with positive feedback regarding motivation, stimulation, and learning with peers. This study demonstrates that blended learning in anatomy practicals, including a flipped classroom and gamification, can enhance the student learning experience and optimize the limited time available for practicals.

**Abstract:**

The use of blended learning strategies is increasingly common in health sciences, including veterinary medicine; however, there are very few descriptions of these methods being applied to practicals. We describe here the application of blended learning based on the implementation of flipped classrooms with collaborative learning and gamification to the 2020–2021 veterinary medicine gross anatomy practicals at CEU Cardenal Herrera University (Spain). Students prepared for the sessions by pre-viewing videos and taking a quiz before the start. The sessions were conducted in small groups where students learned through collaborative work and reviewed their learning with a card game. A small but significant increase was observed when comparing the scores of practical exams of the locomotor apparatus with those of 2018–2019 (6.79 ± 2.22 vs. 6.38 ± 2.24, *p* < 0.05), while the scores were similar (7.76 ± 1.99 vs. 7.64 ± 1.92) for the organ system exams. Students’ responses in a satisfaction survey were mostly positive (>80%) regarding the motivating and learning-facilitating effect of this educational method. Our work shows that the application of blended learning in anatomy practicals based on a flipped classroom and with elements of gamification and collaborative work can be an effective way to improve the learning experience of students.

## 1. Introduction

Higher education in health sciences disciplines is constantly evolving. Advances in technology and the application of new pedagogical approaches are modifying university teaching. In recent years, the emergence of COVID-19 has led to a rethinking of the ways of teaching and to the development of new pedagogical approaches seeking to facilitate the improvement of students’ learning, giving rise to experiences that could then be applied in the post-COVID era [1]. In this context, blended learning (BL) has emerged as a strategy that uses different pedagogical approaches with the aim of modifying some aspects of how students learn to improve their learning experience [2,3,4,5]. The use of combined strategies in anatomy teaching has increased in recent years, with some studies showing the positive effects of using different methodologies [6]. This use of a diversity of e-learning resources, such as quizzes, anatomical demonstrations, computational 3D models, medical imaging, and dissection activities, has been shown to be effective in consolidating anatomical knowledge [7].

It is generally accepted that student-centered approaches and dynamic teaching methods facilitate learning. The flipped classroom (FC), collaborative learning, and gamification have emerged as three methodologies based on these principles, which facilitate active learning and student engagement and ultimately improve student learning [8].

In the FC, the ordinary roles in the learning process are reversed: the teacher acts only as a guide, and the student is the main actor. Flipped teaching is a student-centered approach where information is introduced outside the classroom, allowing more time during class to process the information and practice the content with a variety of active learning strategies [9,10]. The FC provides an opportunity for students to watch and study the course materials on their own time prior to the actual class; once they arrive, they are ready to apply the new knowledge [2,11].

In addition to the FC, peer-assisted or collaborative learning, in which students help each other and learn by teaching, has proven effective in undergraduate medical programmes [12]. More specifically, in anatomy courses, collaborative learning promotes engagement and improves the study and group-working skills of students, moving them from passive consumers to active participants [13].

Gamification experiences, or the use of game design elements in non-game environments to enhance academic performance, have also been used in different health sciences disciplines, reporting generally positive results and improving learning behaviours and learning outcomes [14].

Different BL approaches have been used in anatomy courses, and there is considerable debate about to what extent and how these new methods should be applied and can help to improve students’ learning [7,15,16]. A recent systematic review concludes that the FC is often perceived positively by students and has some advantages, such as increased self-confidence, academic performance, interest, learning activity, interaction, satisfaction, and overall performance in the anatomy curriculum [17]. In relation to collaborative learning, several team learning approaches have been proposed for teaching anatomy [18], and different types of gamification experiences have also been described [19]. In our own experience, the development of a simple card game as a means to consolidate content has been well received by students, who valued the game as enjoyable, challenging, useful for improving their knowledge and understanding of clinical anatomy, and effective for anatomy exam preparation [20].

In this work, we describe a BL teaching experience that combines FC, collaborative learning, and gamification applied to practical sessions of gross anatomy for veterinary students. Similar to in the medical sciences, the use of active learning methods is increasing in veterinary education. A recent international study involving universities in the USA, United Kingdom, and Australia showed that 95% of participants were familiar with the FC technique, although fewer (64%) used it in their teaching [21]. Several studies have published different models of active or BL in veterinary medicine, most of them focusing on clinical or preclinical subjects and generally reporting positive results [22,23]; however, little has been reported in relation to practicals in veterinary anatomy.

With this background, our hypothesis was that introducing elements of flipped learning (so that students had to prepare the content of the practice beforehand), collaborative learning (so that the development of the sessions was cooperative), and gamification (applying a card game that would allow them to review the fundamental aspects of the sessions) to the practicals of veterinary gross anatomy would have positive effects on the learning experience of the students.

## 2. Materials and Methods

### 2.1. Anatomy in the Veterinary Medicine Curriculum

The teaching of anatomy subjects at the CEU Cardenal Herrera University (Valencia, Spain) is divided into three modules, called Structure and Function I, II, and III, during the first three semesters of the Veterinary Degree. The students involved in this study are those enrolled in Structure and Function (SF) I (locomotor apparatus) and II (organ systems), both in the first academic year. The practical sessions of gross anatomy in SF I in the 2020–2021 academic year included 4 sessions of comparative osteology of domestic mammals and 3 sessions of prosections on dog cadavers to study the locomotor apparatus. In SF II, 2 sessions of prosections on dog cadavers (one for the organs of the thoracic cavity and one for the abdominal and pelvic cavities) and 3 sessions of comparative anatomy of organs were carried out. The duration of each session was 2 h for both subjects. A practical exam consisting of identifying a series of anatomical structures was held at the end of each course of SF I (in December) and II (in May).

### 2.2. Participants and Procedure

Students enrolled in the 1st year of veterinary medicine during the academic year 2020/2021 were included in this study. There were 348 students in SF I and 325 in SF II. The students were divided into permanent and stable groups of 12–14 people for each practical. Exclusion criteria: students who did not take the practical examination were excluded from this study.

The study was approved by the Vice-Rectorate for Academic Organisation and Teaching Staff of the CEU Cardenal Herrera University, Ref: PI40A-VV-20.

### 2.3. Videos

Videos showing the anatomical structures to be studied in each session were recorded by the instructors and made available to the students as a basis for preparing the practicals. The videos ranged in length from 3 min to 12′ 30″. They included a description of the main anatomical details in the case of bones and viscera and explained the regional anatomy of the dog on prosections from previously dissected animals. The number of videos included in each practical was variable (between 2 and 5) depending on the content to be covered in each session. Videos were provided for all SF I and SEF II practicals. Students were encouraged to watch the videos prior to the practical as part of their learning process.

### 2.4. Pre-Practical Test

Twelve self-assessment tests were designed (one for each practical session, i.e., seven for SF I and five for SF II). The pre-practical tests were in multiple choice question format (with 4 possible answers, only 1 correct) and contained 5 questions on pictures of the structures explained in the videos (see supplementary material S1 as an example). They were provided via the Internet approximately one week before each practical. Students answered the questionnaire before doing the practical, and they had feedback on their answers immediately after taking the test. Access to the practical was also allowed if the student had not carried out the activity or watched the video.

### 2.5. Anatomy Cards Game

At the end of the practice, the students used a card game with different questions about the practical. Basically, the game consisted of answering questions related to the day’s practical. These questions were organised in 4 groups and appeared on a series of previously prepared cards [20].

### 2.6. Teaching and Learning Approach

#### 2.6.1. Before the Practical

At least one week in advance of each practical, the guide and the videos related to the structures to be identified were made available to the students via the Internet. Students had to study the guide and watch the videos, and then, they had to complete a five-question test via the Internet before the start of the practical. Prior to the implementation of this methodology in 2020–2021, students were provided, as support material, with the guide and a collection of videos that only partially covered the practical content, and no pre-practical quizzes were made available to them. The completion of these tests was taken into account as a component in the assessment of participation in the proposed activities (10% of the total value in SFI and 5% in SF2). The students were allowed to enter the practical regardless of whether or not they had carried out the activity and watched the videos.

#### 2.6.2. During the Practical

In each practical, the students of each stable group (consisting of 12–14 students) were freely subgrouped into small groups of 3–4 students per dissection table to carry out the session (identification and recognition in situ of the corresponding structures). Each session lasted 2 h. Before the application of this methodology in 2020–2021, the teacher explained the structures to be identified for approximately 1 h, and the students used the remaining hour to work on the cadaver or on the models (bone pieces, viscera). In this new approach, students work on the cadaver or models from the beginning, using approximately 1 h 30 min to identify the structures and 30 min to play the self-assessment and knowledge consolidation card game. If the students were not able to identify the structures or had any doubts, an instructor was available to answer their questions. From time to time, this instructor also asked questions directly to the students to verify that the learning was being carried out correctly.

### 2.7. Practical Examination

At the end of each SF I (in December) and II (in May), an oral practical examination was held to assess the knowledge acquired. In the examination session, which was invigilated, the student had to identify 15 anatomical structures. There were 15 sample exams which were randomly distributed among the students at the time of the test. The student, having identified the structure, showed it to the examiner. They had a maximum time of 15 min to complete the test. The exams were similar in the academic years analyzed (2020–2021 and 2018–2019) and carried out by the same examiners in both years.

### 2.8. Questionnaire

At the end of the academic year, a satisfaction survey was presented to the students to find out their level of satisfaction with this teaching system. The purpose of the survey was explained to the students, and they were informed that the data would be used for academic and research purposes only. Participants were also informed that answering the questionnaire was voluntary and that responses were collected anonymously. The main objective of the survey was to determine the overall impact of this methodology on the attitude and learning experience of the students. The questions were designed in such a way that students could assess the effectiveness of the organisation of this course in their learning process. Students were asked about the effectiveness of the pre-recorded videos, the help of other students, the effectiveness in preparing for the practical exam, and about the general consideration of anatomy. For each question, students could choose their level of satisfaction between “strongly agree”, “agree”, “indifferent”, “disagree”, and “strongly disagree”. Students were also given the possibility to express their opinion in qualitative responses. No demographic data were collected. This questionnaire has a Cronbach’s α of 0.77.

The instructors (*n* = 7) also completed a questionnaire asking about the effect of this methodology on improving students’ accountability, the appropriateness of following up on students’ previous work, improving students’ learning, the importance of videos, and the effect on student–teacher interaction. This questionnaire has a Cronbach’s α of 0.60.

### 2.9. Outcomes Comparison

The results of the practical exams obtained by the students were compared with those obtained in the same examinations by students in the 2018/19 academic year to analyse the academic outcomes of this experience. Teaching and examinations were conducted by the same instructors in 2018–2019 and 2020–2021. The academic year 2019/20 was not considered as it was affected by the special measures for COVID-19.

### 2.10. Statistics

Analysis of data included variable scale parameters as mean and standard deviation. Data followed a normal distribution as calculated by Kolmogorov–Smirnov and Shapiro–Wilk normality tests and were compared using Student’s *t*-test. Pearson’s correlation coefficient was calculated to determine the statistical relationship between variables. *p* < 0.05 was considered statistically significant. All analyses were performed using R statistical software, version 4.2.3 for Windows (R Core Team, 2023).

## 3. Results

### 3.1. Students’ Engagement and Academic Results in Locomotor Apparatus Practicals

**Figure 1:**
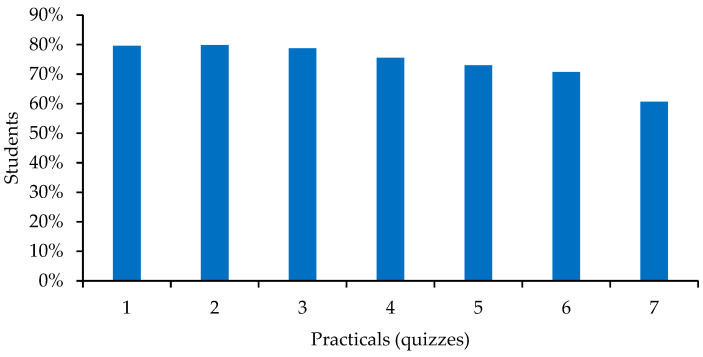
Percentage of students who took the quizzes corresponding to each locomotor apparatus practical.

When analysing the students’ completion of quizzes prior to the practical, results showed that almost all students completed the questionnaires. In total, 74% of the 1803 proposed quizzes were completed. The number of students who completed the questionnaires, which was fairly constant (between 75% and 80%) for the first ones, decreased slightly towards the middle of the course and markedly in the last quiz proposed, which was completed by 61% of them (Figure 1).

Almost half (*n* = 163, 47%) of the students took all the quizzes and 20% (*n* = 68) only left one undone. On the opposite side, 48 students (14%) did none (Table 1).

We then examined the correlation between having completed the quizzes and the practical exam marks. The students’ final score in the anatomy practical exam was 6.79 ± 2.22 out of 10. We found a direct and discreet relationship (0.29, *p* < 0.001) between the number of quizzes completed by the students and their marks in the practical exam. We then analysed whether the opposite groups of students, i.e., those who had completed all the quizzes and those who had completed none of them, differed in their marks in the practical exam. Students who had completed all seven quizzes obtained a mean score of 7.67 ± 1.98 compared to those who had completed none, who scored 5.98 ± 1.98. This difference was very highly significant (*p* < 0.001).

Comparing these results with those obtained in the academic year 2018–2019 (the 2019–2020 academic year was not considered due to the special conditions of its teaching by the appearance of COVID-19), we found that academic results in 2020–2021 (6.79 ± 2.22) were slightly but significantly higher than in 2018–2019 (6.38 ± 2.24; *n* = 353, *p* < 0.05).

### 3.2. Students’ Engagement and Academic Results in Organ System Practicals

**Figure 2:**
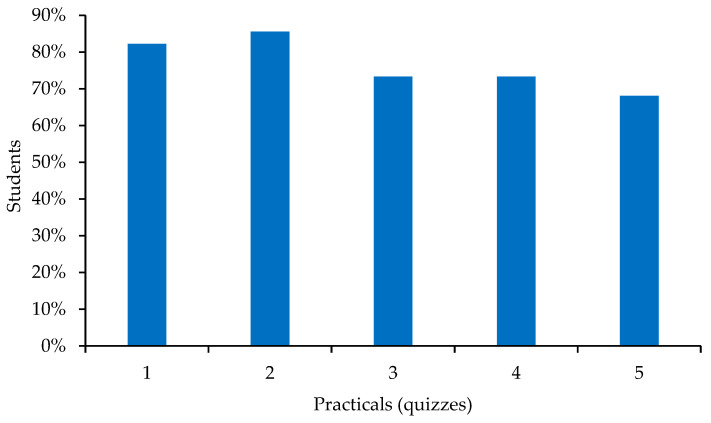
Percentage of students who took the quizzes corresponding to each organ systems practical.

In total, 78% of the 1273 questionnaires related to organ systems were completed by students. The first two questionnaires were completed by more than 80% of the students, and thereafter there was a very slight but steady decline in the number of students taking the tests (Figure 2).

The number of students completing the questionnaires in the organ systems practicals was slightly higher than in the locomotor system practicals, with more than half of the students (*n* = 178, 55%) completing all the questionnaires and only 23 (7%) not completing any (Table 2).

The students’ score in the final organ systems practical examination was 7.76 ± 1.99. A positive correlation (0.33, *p* < 0.001) was observed between the number of quizzes completed by the students and their marks in the practical exam. Comparing the scores of students who had completed all quizzes (8.31 ± 1.81) with those who had completed none of them (6.23 ± 2.04), a very highly significant difference was again found (*p* < 0.001). However, no difference was found, in this case, when comparing the marks for the organ systems practical exam in the year of this study (7.76 ± 1.99) and in 2018–2019, wherein students obtained 7.64 ± 1.92 (*n* = 372).

We next compared the percentage of students with low performance (those obtaining less than 5 out of 10 in the practical exam) in both SF I and SF II. The percentage of students with low performance was reduced in SF I (19% in 2020–2021 vs. 29% in 2018–2019), while in SF II, there was no change (10% in 2020–2021 vs. 8% in 2018–2019).

### 3.3. Students’ and Instructors’ Satisfaction Survey

**Figure 3:**
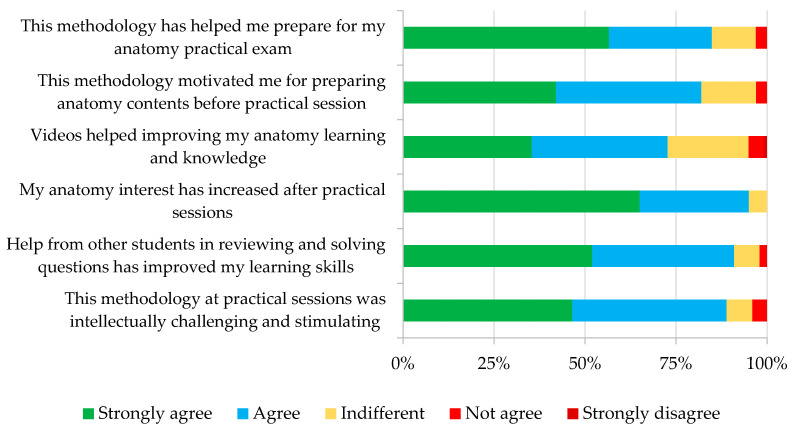
Results of the global satisfaction survey carried out by the students.

A satisfaction survey was provided to the students at the end of the course, which was completed by 167 students. The majority of students answered positively to the proposed questions, as more than 80% of respondents agree or strongly agree with most of the proposed questions, with “*My anatomy interest has increased after practical sessions*” being the highest percentage (95%) and “*Videos helped improving my anatomy learning and knowledge*” the lowest (72%) (Figure 3).

The students also had the opportunity to give their opinion, and 78 of them expressed their qualitative comments on the system. Most of them were positive, but in some cases, they suggested modifications in the method or in the quality of the materials provided. Some examples of the students’ opinions are as follows:

“*It would be nice to have access to the videos and tests from the beginning of the course and have the ability to do the tests multiple times*”.

“*I think videos are great bonus tools, as well as doing a test before each practical. But i prefer very much to be taught by anatomy teachers during the practical itself. So basically, watch vids, do the quizzes and then be taught by the teachers during practicals to avoid confusion. Even though they’ve always been available in case of doubts;)*”.

“*I would like to say that without the videos, I would never have obtained the grade I had at the anatomy practical exam. I really appreciate the videos but I can say that some of them didn’t really follow the guide. The cards are really good to train ourselves during anatomy sessions. It would be great that they last longer and students helping us during the practicals is also very useful.*”

**Figure 4:**
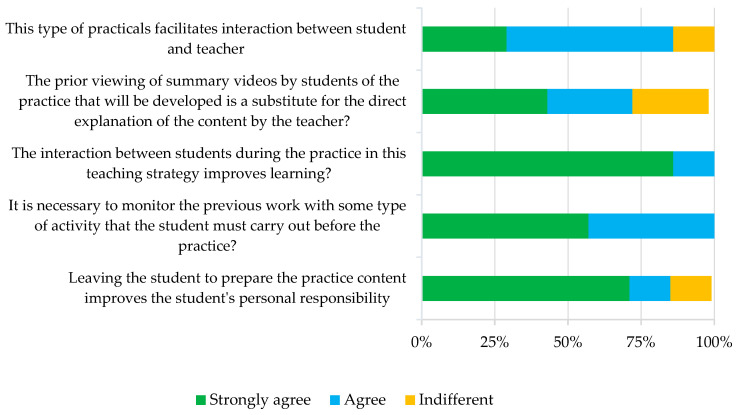
Results of the global satisfaction survey carried out by the teaching staff.

Additionally, a questionnaire was also completed by the teaching staff (*n* = 7). The results indicate that, in the opinion of the staff, this methodology is particularly positive for improving the personal responsibility of the students and also highlights the convenience of checking the students’ previous work with questionnaires before doing the practicals. On the other hand, although mostly positive, there was a diversity of opinions as to whether the videos can replace the direct explanation of the content by the teacher and whether the system increases teacher–student interaction (Figure 4).

## 4. Discussion

### 4.1. The Usefulness of Blended Learning in the Teaching of Anatomy and the Academic Outcomes of the Students

Many different approaches are used to teach anatomy, and no single teaching tool has been found that meets all curriculum requirements [24,25,26]. In this regard, the application of new teaching methodologies has been described for both medical and veterinary anatomy, and ultimately, all the different changes that have occurred in recent years, including those resulting from the emergence of COVID-19, have promoted the need for adaptation and the development of new learning models [27,28,29,30].

Among the multiple forms of training, some authors consider that the best way to teach anatomy is by combining multiple pedagogical resources that complement each other, maximising interactive learning, and using time-saving materials that help students learn gross anatomy effectively and efficiently [29,30,31].

We describe here the transformation of traditional hands-on teaching into a BL approach in veterinary anatomy practicals. This change basically consisted of transforming sessions where a large part of the time was occupied by teacher explanation into active learning sessions, integrating flipped teaching, collaborative learning, and gamification. The results indicate that this switch was highly appreciated by the students and had some beneficial effect on academic results.

Different methods can make up a BL experience in anatomy. In our study, the FC is the central element, which is closely linked to students’ cooperative work throughout the sessions and to a gamification element consisting of a card game designed to self-assess and reinforce knowledge [20]. Our results are in line with those of other authors who have reported the beneficial effects of BL in human anatomy teaching and show that active learning strategies are appreciated by students. Medical students preferred the hybrid, interactive, and student-centred learning method for practicals as a more effective system for understanding anatomy than face-to-face or online methods [32]. In another work, students appreciated the pre-class activities of this kind of learning, as they facilitated their engagement and concentration during class, thus increasing their academic performance and engagement [33]. In this regard, it has also been shown that veterinary anatomy students also favour active learning classes compared to traditional classes [34].

### 4.2. The Implementation and Usefulness of Flipped Classroom

Pre-class work is essential in FC pedagogies. Preparing courses by studying the material provided allows for better use of class time, creating time for integrative group activities during the session [35]. In this model, students have more responsibility for each laboratory experience, which can lead to more effort, but also to more satisfaction and confidence. While most FC approaches are applied to lecture-based courses, our results show that FC could also be applied to practicals. In our study, most of the students adequately prepared for the practicals before attending the sessions. In the preparation of the sessions, the use of videos is very important. Video as a support material for anatomy lectures is considered a very useful resource in a condensed curriculum scenario [36,37], and we have shown that their use can help decrease anxiety and enhance students’ learning experience [38]. In our study, students considered these videos as important elements in the preparation of the practicals, although, in some cases, students requested more detailed documents. This would explain why the degree of satisfaction was not as high as in other questions of the survey. Although we do not have data on the video visualizations, the fact that many students completed the quizzes before the practicals suggests that most of the students prepared themselves thoroughly before attending the practicals. The number of completed quizzes exceeded 75% in the first sessions, but there was a 15–20% reduction in the number of tests completed towards the end of the semester. This decrease is consistent with Greene’s [37] study, which showed a significant drop over the year in the total viewing of videos provided to review structures identified in lab sessions throughout an organ systems course. Likely, the decrease in completion of the quizzes was due to the feeling that time was not sufficient at the end of each semester, as reported in other studies [36,37,39]. At the end of the semester, there is generally a greater workload for the student; obviously, this depends on the organisation of the curriculum, but it is common for these dates to coincide with imminent exams, the preparation of which requires extra time and creates stress for students that can cause them to drop some activities. It is interesting to note that there is a correlation, though not very high, between having carried out these activities and academic outcomes. However, if we compare the results obtained by the students who completed all the tests with those who did not do any, the differences are highly significant in both topics, indicating the effectiveness of active involvement in this type of teaching.

Different results have been published on the effect of FC on academic scores in several disciplines of health sciences. Most of the studies report a slight improvement in test scores, while others show neutral results. Tune et al. [40] stated improvements of around 11–12% in the results of cardiovascular, respiratory, and renal physiology tests. Street and colleagues (2015) described a slight improvement, at the limits of the statistical significance, in a preclinical physiology course [41]. Better results after the implementation of FC have also been reported in practical examinations of dental students [42], in a pharmacotherapy course for pharmacy students [43], in an advanced physiology course for pharmacy students [44], and in physiology courses for health sciences students [45].

In other cases, the development of FC did not produce improvements in the performance of the students. No differences in grades or level of satisfaction were shown in an FC for neuroanatomy [46], and learning gains were not significantly different between a flipped biochemistry course and a traditional lecture [47]. However, in this last report, student perception of learning gains did differ and indicated a higher level of satisfaction with the flipped lecture format [47]. Indeed, students’ perceptions about the effectiveness of FC are not necessarily accompanied by improvement in academic performance. This was also shown in a study performed in an introductory epidemiology class on masters-level graduate students [2].

A partial improvement has also been reported. Pharmacy students’ performance in a flipped teaching pilot on cardiac arrhythmias improved in two of the three classes [48], and in a preclinical course (foundations on animal health) of veterinary medicine, FC cohorts performed well in the final exam but not in multiple-choice tests [23].

Similar results have been reported in studies specifically related to anatomy. A recent review shows that most FC experiences in anatomy report better exam scores than traditional courses, although there is also some work reporting a neutral effect [17]. Elzainy and Sadik [49] reported that FC medical students scored higher than traditional classroom students in anatomy theory classes and had lower absenteeism. Moreover, a small but significant improvement has been demonstrated in the histology exam scores of FC students compared to the control group [50], and nursing students instructed with the FC method on anatomy and physiology of the respiratory system scored higher on the final exam than the control group [51]. Finally, results reported by Yang and colleagues [52] and Elzainy and Sadik [49] on different human anatomy experiences for medical students pointed in the same direction.

As noted above, some anatomy-specific studies also describe partial improvements or neutral results from the application of FC. Fleagle et al. reported that there was no change in mean scores on the first and second lab exams, but higher scores on the final lab exam for dental gross anatomy students [35], and in another study, FC students also performed better than lecture hall students on analysis items, but there was no difference in performance between students in the two groups for knowledge [53]. Our results are close to the latter works, as the students in the experience we present here obtained a slight but statistically significant improvement in the practical examination of the anatomy of the locomotor system, while there was no effect in the examination of the anatomy of organ systems and body cavities.

### 4.3. The Role of Collaborative Learning and Gamification in a Model of FC

FC and peer learning are closely related since FC provides more opportunities for students to interact effectively and learn from their peers [2]. It has been shown that cooperative learning deepens student understanding but also offers them an opportunity to practice the generic skills in veterinary anatomy dissection courses, enhancing students’ comprehension of the subject matter and encouraging them to share responsibility for their learning [54]. Peer-assisted learning also had positive results in a study on medical students, which appreciated most aspects of this method and obtained higher scores in osteology [55]. In this sense, it has been shown that combining flipped teaching and team-based learning results in positive outcomes in a physiology course [44]. On the basis of a different experimental design, Laakkonen and Muukkonnen [56] also pointed out the importance of collaboration between students to support mutual learning in gross anatomy courses of veterinary medicine. Among the diversity of peer-assisted learning methods, our model is based on collaborative work between students, where, organised into small groups, they received help from other students who solved their doubts in the first instance and in reviewing the content. In fact, this feature was one of the most valued aspects by the students, emphasizing the importance of collaborative work in this type of teaching. Interestingly, a positive outcome has been reported when students work cooperatively rather than when peer interaction was absent [9]; it has also been shown that the combination of e-learning, peer teaching, and FC significantly improved the average score in a physiology laboratory course for nursing students [57].

The development of the card game that ends each practical session in our model was also carried out in a collaborative environment [20] in a manner wherein everyone learns, regardless of the outcome of the game. In this sense, positive student outcomes have been reported in different gamification experiences in anatomy and related disciplines [58,59], reinforcing the positive effects of cooperative learning. Similar to other active approaches, the use of game-based methods is increasing in anatomy, and although the research concerning game-based anatomy learning is relatively limited, gamification seems to have a positive impact on different aspects of students’ learning [16].

### 4.4. The Students and Staff Perceptions of Active Learning

Beyond the academic results, one of the advantages of the different BL models is that they are appreciated by the students as they improve their learning experience. In our work, as previously indicated, students rated the interaction with other students very positively and found the system motivating and challenging, and it increased their interest in anatomy. In the flipped model, students have more responsibility, which can increase effort but also satisfaction and confidence [35]. Active learning in anatomy demonstrations has been shown to be a preferred method by students [32], and perception surveys revealed students’ enthusiasm for pre-class activities, leading to better performance in class with increased engagement with peers and teachers [60]. The positive perception of students toward active learning has been highlighted in numerous works ([60] and references therein). These results are also consistent with those shown in a systematic review showing that FC is generally perceived positively by students, increasing self-confidence, academic performance, interest, learning activity, interaction, satisfaction, and overall performance in the anatomy curriculum [17]. It is possible that this new system would entail a little more workload. This aspect was not explored, but the overall high degree of student satisfaction suggests that it was not an excessive effort.

Regarding the opinion of the staff, most of them were positive about the benefits of this method, since they all considered that it improves personal responsibility and ultimately student learning, but it is also important to point out the convenience of monitoring the work as a means of control of the preparation of the practical. This type of opinion is not always generalizable, and it has been described that, sometimes, teachers are more reluctant to implement this type of teaching than students. In this regard, it has been described that teachers sometimes consider that students do not prefer FC to traditional teaching and are not sure of the students’ response to FC, in contrast to the positive views about this method in the student satisfaction survey [33].

### 4.5. Limitations of the Study

This teaching system was installed in the anatomy practicals for all students as a consensual teaching decision accepted by the University, so there is no control group in the same year. For this reason, the comparisons in the academic results have been made with those of the 2018–2019 academic year, the last one in which the presence of COVID-19 did not alter the teaching strategy.

On the other hand, the organisation of the teaching did not make it possible to follow the viewing of the videos.

Finally, a demographic study was not carried out, so it is not possible to know whether such factors influenced the results.

## 5. Conclusions

Our study shows that the development of an FC system in veterinary anatomy practices can have a beneficial effect on academic results. Moreover, it is well appreciated by students who positively value cooperation with other students to solve problems and consider this a highly motivating methodology. While most FC experiences are applied to lectures, our approach is aimed at practical gross anatomy classes. According to our experience, the application of this type of teaching organisation allows for better use of time, which is increasingly reduced in many anatomy curricula, and provides a good teaching experience for students and staff. Learning based on FC with a strong cooperative component among the students and completed with some gaming element, such as the work that we present here, can be applied, with the necessary adaptations, in a general way, both in veterinary medicine as well as in the different disciplines of health sciences that involve the practice of veterinary and human anatomy.

## Figures and Tables

**Table 1:** Number of quizzes completed by students in the locomotor apparatus study.

Number of Quizzes Completed	0	1	2	3	4	5	6	7
Number of students (%)	48 (14%)	8 (2%)	7 (2%)	11 (3%)	16 (5%)	27 (8%)	68 (20%)	163 (47%)

**Table 2:** Number of quizzes completed by students in the organ systems practicals.

Number of Quizzes Completed	0	1	2	3	4	5
Number of students (%)	23 (7%)	15 (5%)	18 (6%)	32 (10%)	59 (18%)	178 (55%)

## Data Availability

The data presented in this study are available on request from the corresponding author.

## Supplementary Materials

The following supporting information can be downloaded at: https://www.mdpi.com/article/10.3390/ani13101666/s1, S1: Example of a question in a pre-practical quiz.
