# Supplementary figures and images for: Application of Blended Learning to Veterinary Gross Anatomy Practical Sessions: Students’ Perceptions of Their Learning Experience and Academic Outcomes
Source: Animals (Basel). 2023 May 17;13(10):1666. doi: 10.3390/ani13101666 (PMC10215638; doi:10.3390/ani13101666)

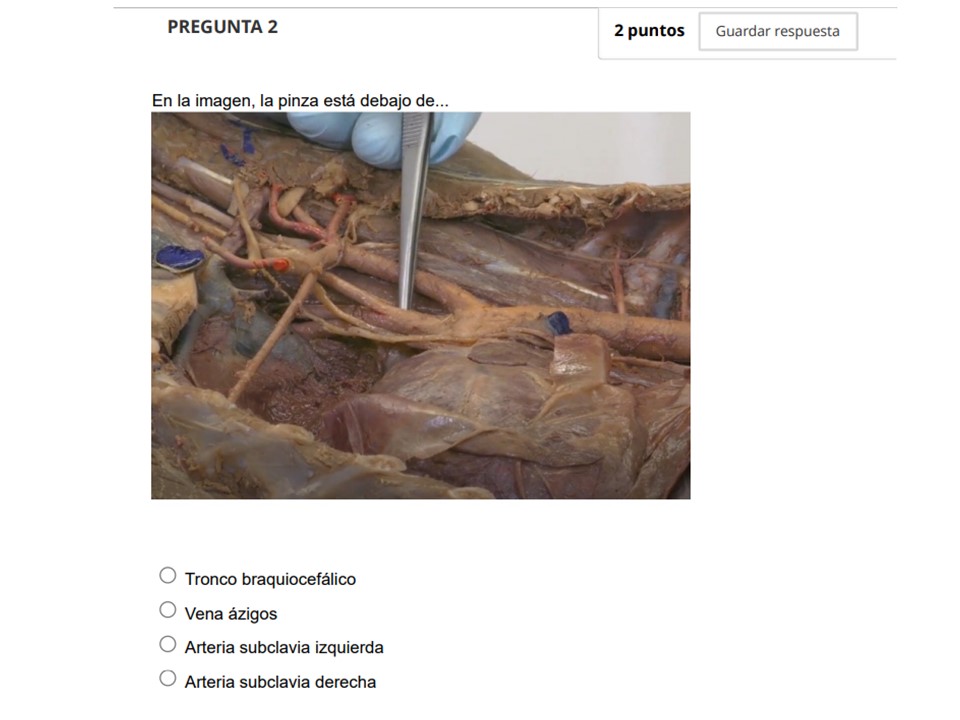

Supplement: Supplementary file 1 [file animals-13-01666-s001.zip › animals-2355903-supplementary.jpg]
